# The Last Whistle: Unveiling the Relationship between the Career Path and Retirement Age in Professional Soccer

**DOI:** 10.5114/jhk/194996

**Published:** 2025-05-29

**Authors:** André Rebelo, Diogo S. Teixeira, Diogo Monteiro, Ricardo Monteiro, Bruno Travassos

**Affiliations:** 1 CIDEFES, Centre for Research in Sport, Physical Education, Exercise and Health, Lusófona University, Lisbon, Portugal.; 2 COD, Center of Sports Optimization, Sporting Clube de Portugal, Lisbon, Portugal.; 3 ESECS—Polytechnic University of Leiria, Leiria, Portugal.; 4 Research Centre in Sport, Health and Human Development (CIDESD), Vila Real, Portugal.; 5 Department of Sport Sciences, University of Beira Interior, Covilhã, Portugal.; 6 Portugal Football School, Portuguese Football Federation, Oeiras, Portugal.

**Keywords:** transition support, sports psychology, early career, discontinuation stage, career guidance

## Abstract

This study explores the relationship between Career Indicators (CIs) and the retiring age of Portuguese soccer players, focusing on the impact of early career experiences and career discontinuation stages. Data were analyzed from retired Portuguese soccer players registered on a private digital platform, using specific CIs related to different stages of their athletic careers. The analysis involved a two-level mediation model incorporating various CIs. Significant direct effects emerged with ‘the number of seasons as a youth player’ and ‘the number of seasons as a youth player in top 3 clubs’ on ‘retiring age’. Additionally, a strong association was identified between ‘discontinuation stage length’ and ‘retiring age’. The findings emphasize the importance of effectively managing career discontinuation stages and proactive career planning. Consequently, comprehensive support programs offering resources and guidance for career transitions are recommended for soccer players. The study underlines the key role of stakeholders in enabling smoother transitions into retirement.

## Introduction

In professional sports, and particularly within soccer, career planning is paramount. Proactive career planning allows one to map out milestones, anticipate challenges, and set goals that align with the athlete's aspirations and potential (Stambulova et al., 2021). It encompasses not only the progression of the player on the field, but also their educational, social, and financial strategies during the career and after career termination. Well-structured career planning ensures that athletes are equipped with the necessary skills and knowledge for both their time in professional sport and their life beyond it. This forward-thinking approach aids players in maximizing their potential, prolonging their playing years, and ensuring smoother transitions from youth to career termination (Stambulova, 2010).

In the context of soccer, these issues become more pronounced. Soccer players typically start their careers at a young age, which often leads to a compromise on their education (Curran, 2015). This early specialization and the lack of focus on education can limit their career options post-retirement. Furthermore, the relatively short span of a professional soccer career, combined with high income during playing years, can lead to financial mismanagement, thereby causing financial difficulties after retirement (Curran, 2015).

Moreover, the prevalence of injuries in soccer can lead to long-term physical health issues, further complicating career termination (Roos, 1998). Athletes’ career termination is increasingly recognized as a complex process that requires careful management across the lifespan to mitigate potential negative repercussions on the former athletes’ life (Baillie and Danish, 1992; Knights et al., 2019). The transition from active professional sport to retirement is not merely an event, but a multifaceted process that impacts multiple aspects of athletes' lives, including mental and physical health, as well as social and economic dimensions (Park et al., 2013).

Psychologically, athletes may struggle with a sense of identity loss, depression and a lack of direction after their professional sports careers end (Allison and Meyer, 1988). They spend their formative years cultivating a sense of self that is closely tied to their athletic pursuits, and this sudden shift can lead to feelings of emptiness and loss of purpose (Werthner and Orlick, 1986). The social aspect of retirement is equally challenging. Athletes are often accustomed to a highly structured environment with a strong social network of coaches, teammates, and support staff. The abrupt departure from this environment can lead to feelings of isolation and difficulty in building new social networks (Cosh et al., 2013).

Therefore, understanding the factors that influence the career length and retirement age of professional soccer players is crucial in the sports science and performance domain. The complexity of these variables and the myriad of potential influences suggest that career length and retirement age are likely multi-faceted issues. Recent evidence suggests that various career indicators (CIs), such as the number of seasons played at different levels, the age of first registration as a senior player or even the age of achieving the best result, may have a significant impact on a soccer player’s career length and retirement age (Monteiro et al., 2020, 2022).

Traditionally, players’ career progression has been classified into three stages: initiation/development, mastery, and discontinuation (Wylleman, 2019). Each of these stages carries its own unique set of characteristics, challenges, and achievements, all of which may contribute to a player's career length and retirement age (Wylleman, 2019; Wylleman et al., 2013). In the initiation/development stage, the number of seasons spent as a youth player and at top-level clubs may provide the foundational skills and experience necessary for a successful senior career (Wylleman, 2019; Wylleman et al., 2013). In the mastery stage, the player's performance and achievements in senior leagues may dictate their career longevity (Wylleman, 2019; Wylleman et al., 2013). Finally, in the discontinuation stage, the ability to maintain performance levels and manage physical health could be critical determinants of the length of this stage and, subsequently, retirement age (Wylleman, 2019; Wylleman et al., 2013).

A recent study began to unveil the direct relationships between various CIs and the retirement age of professional soccer players (Monteiro et al., 2022). This pivotal research highlighted the potential existence of indirect effects between these variables, revealing a gap in the current understanding and hinting at deeper, underlying mechanisms that influence career trajectories. Building on these findings, the present study delves deeper into the mechanisms underlying the relationships identified in the study by Monteiro et al. (2022). The aim was to analyze both the direct and indirect effects of CIs at different career stages on the retirement age of professional soccer players in Portugal. By investigating these mechanisms, we aimed to offer a richer understanding of career length and retirement age in professional soccer.

The exploration of these relationships is paramount for a holistic understanding of the career dynamics of soccer players and can directly benefit players, coaches, and clubs alike. Professional soccer clubs, in particular, stand to gain from these insights as they can help manage athletes more effectively, especially during the discontinuation phase, where career instability is more pronounced. A well-managed player can not only prolong their career, but also prepare for post-career opportunities, such as investing in education and transitioning into leadership roles within clubs. Additionally, clubs can play a crucial role in supporting players through career management programs that prepare them for retirement and mitigate post-career uncertainties. Therefore, the objective of this study was to test a two-level mediation model where CIs served as parallel mediators (first level) and series mediators (second level). The central question addressed in this paper was: what are the direct and indirect effects of CIs at different career stages on the retirement age of professional soccer players in Portugal?

## Methods

### Sample

A total of 3,467 participants were confirmed eligible and included in the study. Participants in the study were all retired Portuguese soccer players with an average age of 32.70 ± 4.27 years at retirement and an average of 17.51 ± 4.89 years spent as professional soccer players. Participants were selected based on the availability of career data from their youth level to the end of their careers. This retrospective cohort longitudinal observational study design enabled the tracking of their career paths, capturing their initiation and development stages, along with mastery, and discontinuation stages. The sampling was non-probabilistic and for convenience, as participants were included based on the completeness and availability of their career data. The data related to these stages were divided into several CIs, providing a multifaceted view of each athlete's career. Data were collected from the www.zerozero.pt platform concerning retired Portuguese soccer players who played between 1960 and 2018. From this pool of potential data, the eligibility of data points was examined based on the requirement for complete career tracking from the youth level, i.e., the first youth registration, to the end of the player's career, i.e., retirement. The “data points” referred to key career milestones such as the number of seasons played, the clubs they played for, and age at significant career events.

### Variables

The key variables in this study were divided into outcomes, exposures, predictors, and effect modifiers. The primary outcome variable was the retiring age, representing the age at which a soccer player officially ended their professional career. The exposure variables included the number of seasons as a youth player and the number of seasons as a youth player in top 3 clubs. The predictors encompassed multiple CIs across the mastery stage of the players' careers. These predictors were as follows: the age of the first registration as a senior player, the number of seasons played as a senior player, the number of seasons played as a senior player in top 3 clubs, the total number of games as a senior player, and the age of the last best result achieved. The discontinuation stage length acted as a second stage mediator as proposed by Monteiro et al. (2020).

### Procedures

In line with the Declaration of Helsinki of 1964, ethical approval was obtained from the Research Centre in Sport, Health and Human Development (protocol code: UIDB/04045/2020; protocol date: 01 January 2020). The study was carried out in several stages to ensure a throughout analysis of the relationships between CIs and the retiring age of professional Portuguese soccer players. Initially, data were systematically retrieved from the www.zerozero.pt digital platform, focusing on retired Portuguese soccer players who played between 1960 and 2018. This initial step involved filtering the database for players with complete career data, from the youth level through to the end of their professional careers.

Upon identifying eligible participants, data extraction commenced, focusing on variables relevant to the study’s objectives. These variables included both the players' career milestones and performance metrics, such as seasons played at youth and senior levels, the number of games played, and ages at significant career stages. Following data extraction, the study proceeded with a descriptive and correlation analysis to assess the basic characteristics of the dataset and examine the relationships between independent and dependent variables. This step was crucial for verifying the assumptions underlying the statistical models to be employed and for informing the mediation analysis framework.

### Statistical Methods

Descriptive and correlation analyses were performed for analysis assumptions. For mediation purposes recommendations of Hayes (2018) were followed. The SPPS macro PROCESS v. 3.5 was used and model 80 was chosen in order to test the indirect effects and interactions of proposed mediators’ in the relation between the independent and dependent variables. This model allowed to estimate the direct effect of the independent variables (A: Number of seasons as a youth player; B: Number of seasons as a youth player in top 3 clubs) on the dependent variable (retiring age), and the indirect effects through multiple mediators structured both in parallel (age at first registration as a senior player; number of seasons as a senior player; number of seasons as a senior player in top 3 clubs; number of total games as a senior player; last best result age achieved) and in series (discontinuation stage length). The hypothesized model allowed the estimation of individual indirect effects through the first level of mediators (parallel) independently of the second level of mediation (series), and posteriorly, the estimation of the indirect effects with all mediators’ interaction, thus allowing distinct mediators’ analysis in the total model effect.

Given the moderate correlation scores between the independent variables (r = 0.411, *p* < 0.001), a decision was made to maintain and test both of the variables in the same model, as suggested by Hayes (2018), thus allowing all theoretically proposed variable interactions analysis (Hayes, 2018). A bootstrap with 10,000 samples and a 95% confidence interval (95% CI) estimate was calculated for significant indirect effects detection (if 95% CI did not encompass zero) (MacKinnon et al., 2004).

## Results

Descriptive statistics and bivariate correlation results of the player's characteristics and variables under study are presented in Table 1 and Table 2, respectively. Generally, patterns of positive correlation emerged among all variables. The exceptions related to ‘discontinuation stage length’, which was negatively associated with most variables (except with ‘the number of seasons as a senior player’), and ‘age at first registration as a senior player’, also negatively associated with the majority of the variables (except ‘the age of the last best result achieved’ and ‘retiring age’).

**Table 1:** Descriptive analysis of the players characteristics and variables studied.

	Min	Max	M	SD
Number of seasons as a youth player	0.0	19.0	2.6	2.6
Number of seasons as a youth player in top 3 clubs	0.0	10.0	0.5	1.3
Age at first registration as a senior player	15.0	19.0	17.9	0.7
Number of seasons as a senior player	5.0	27.0	14.2	4.3
Number of seasons as a senior player in top 3 clubs	0.0	18.0	0.5	1.9
Number of total games as a senior player	1.0	919.0	93.0	125.7
Age at which the last best result was achieved	16.0	44.0	26.9	5.1
Discontinuation stage length	0.0	21.0	5.5	4.6
Retiring age	21.0	45.0	32.7	4.3

**Table 2:** Correlation analysis of the variables under study.

	1		2		3		4		5		6			7	8	9
1. Number of seasons as a youth player	1															
2. Number of seasons as a youth player in top 3 clubs	0.411**		1													
3. Age at first registration as a senior player	−0.001		−0.013		1											
4. Number of seasons as a senior player	−0.052**		−0.023		−0.137**		1									
5. Number of seasons as a senior player in top 3 clubs	0.052**		0.240**		−0.128**		0.147**		1							
6. Number of total games as a senior player	0.023		0.188**		−0.117**		0.403**		0.555**		1					
7. Age at which the last best result was achieved	−0.035*		0.001		0.070**		0.536**		0.248**		0.452**			1		
8. Discontinuation stage length	−0.019		−0.032		−0.081**		0.289**		−0.172**		−0.172**			−0.601**	1	
9. Retiring age	−0.057**		−0.042*		0.019		0.936**		0.101**		0.330**			0.522**	0.358**	1

^*^ *p* < 0.05; ** *p* < 0.01

**Figure 1:**
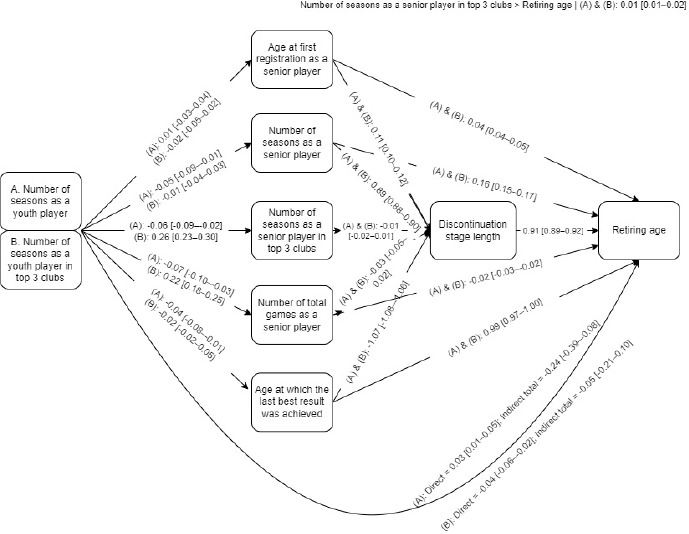
Multiple mediation model analysis for the hypothesized paths under study.

Regarding mediation analysis procedures (Figure 1), several relevant interactions emerged. First, significant direct effects were detected both with ‘the number of seasons as a youth player’ (A) and ‘the number of seasons as a youth player in top 3 clubs’ (B), although with low association scores (A: β = 0.03 [0.01–0.05]; B: β = −0.04 [−0.06–−0.02]); total indirect significant effects only emerged in the independent variable ‘the number of seasons as a youth player in top 3 clubs’ (β = −0.24 [−0.39–−0.08]). In the first level of mediation analysis (parallel), the most relevant interactions were related to the independent variable ‘the number of seasons as a youth player in top 3 clubs’, ‘the number of seasons as a senior player in top 3 clubs’ (β = 0.26 [0.23–0.30]) and ‘the number of total games as a senior player’ (β = 0.22 [0.18–0.25]).

Between the first and the second level (series) of mediators’ interactions, significant and relevant scores were found between ‘the number of seasons as a senior player’ (β = 0.89 [0.88–0.90]) and ‘the age of the last best result achieved’ (β = −1.07 [−1.08–−1.06]) with discontinuation stage length. As for the first level of mediators and the dependent variable interactions, most relevant and significant effects were detected between ‘the number of seasons as a senior player’ (β = 0.16 [0.15–0.17]) and ‘the age of the last best result achieved’ (β = 0.99 [0.97–1.00]).

Regarding the first and the second level of interactions among combined mediations , the ‘discontinuation stage length’ presented a significant association with the dependent variable (β = 0.91 [0.89–0.92]).

## Discussion

The present study aimed to provide an examination of the relationships between various CIs across career stages and the retirement age of professional soccer players, through a two-level mediation model where CIs served as parallel mediators (first level) and series mediators (second level). As expected, the results revealed direct and indirect effects of CIs at different career stages on the retirement age of professional soccer players in Portugal. Such information can be used to support long-term planning and to manage player's career effectively.

Previous research revealed that the path athletes traverse throughout their careers plays a crucial role in determining their retirement age and its success (Knights et al., 2019; Park et al., 2013; Stambulova et al., 2021). According to the classification of the stages of players’ career development, the results revealed significant correlations between the retirement age of Portuguese soccer players and the CIs from both the youth and senior stages of their careers. It reinforces the need to consider the players’ career as a path in which the long-term planning and decision should be informed and sustained based on reliable information that supports an effective career management. In fact, career indicators can be used as a guide or assessment tool to inform career decisions as well as retirement, but they should be considered within the context of the player's circumstances with reference to the general tendencies (Park et al., 2013). For example, as youth, the practice in a top 3 club and the number of years of such practice, seem to be very important to sustain the longevity of an athlete’s career and the age of retirement. Such information is of high importance for parents and for youth athletes that prospectively plan in a conscious way their career and particularly each normative transition. This could also be of particular interest to youth players that need to choose between clubs according to their level and conditions to sustain their dual career over their development stage.

Accordingly, the impact of a player's tenure in top 3 clubs during their senior career stood out. It was observed that having a greater number of seasons played in such high-ranking teams and a higher number of total games as a senior player had substantial effects on prolonging the player's career. This may be due to the acquisition of advanced skills, increased resilience, enhanced opportunities, and exposure that playing in top-tier clubs might offer, which could extend the player's competitive career (Stambulova et al., 2021). Thus, the level of players’ career seems to be of paramount importance considering its impact on the age of retirement and the length of a player's career.

In line with that, a strong correlation was observed between ‘the number of seasons played as a senior’ and ‘the discontinuation stage length’, demonstrating that the length of a player's career at the senior level significantly impacts the duration of their career transition. More important than that, the ‘discontinuation stage length’ exhibited a substantial correlation with ‘the age of the last best result achieved’. Aligned with previous literature, this suggests that achieving notable career milestones later in their career might be linked with a shorter discontinuation stage length (Sinclair and Orlick, 1993). This could potentially be due to these players' ability to leave the sport on a high note, satisfying their career ambitions, and hence experiencing a smoother transition into retirement. Conversely, it could also indicate a sudden end to their career following peak performance, leaving them less time to plan and adjust for life after soccer. Thus, the variables ‘the number of seasons played as a senior’ and ‘the age of the last best result achieved’ could be considered important for the identification of ‘the discontinuation stage length’ and be used by athletes to evaluate the stage of their own career as well as inform them about possible decisions related to their career.

The strong positive association between ‘discontinuation stage length’ and the dependent variable of retiring age, suggests that the length of the discontinuation stage is significantly related to the age at which soccer players retire. Essentially, this means that the longer the discontinuation stage is (i.e., the longer period players spend transitioning out of their soccer careers), the later they tend to retire. This could be due to a variety of factors. Players who have a longer discontinuation stage might spend more time preparing for the end of their active soccer careers, perhaps by acquiring new skills or exploring post-soccer career opportunities (Park et al., 2013). This preparation could enable them to extend their soccer careers, perhaps by reducing the stress or uncertainty associated with retirement and allowing them to focus more on their current playing responsibilities. On the other hand, a longer discontinuation stage might also reflect difficulties in transitioning out of a soccer career. Some players might find it challenging to leave a sport that has defined a significant part of their lives and identities. Consequently, they might delay their retirement and play at a non-professional level, leading to a longer discontinuation stage (Cecić Erpič et al., 2004; Dimoula et al., 2013). These results emphasize the importance of providing sufficient resources and support to players as they navigate the discontinuation stage (Holding et al., 2020). Effective career transition programs could potentially enable players to retire at an optimal time, balancing their immediate desires to continue playing with their longer-term life and career goals. Notwithstanding, it is important to consider the potential multiple pathways and interactions among these factors, indicating a complex, multifaceted process that warrants further investigation. Also, future studies could delve deeper into understanding the influence of other potential confounding factors such as injuries, financial stability, and personal circumstances, which may contribute to the retirement decision.

## Limitations

While this study yielded valuable insights, it is crucial to acknowledge its limitations. Primarily, the research relied solely on public data from a private digital website platform. This reliance may have introduced some degree of bias or error, as the accuracy of the study depends heavily on the correctness of the website's data, which might not be as rigorous or thorough as data collected directly from primary sources or official databases. The moderate correlation observed between the independent variables suggests potential collinearity, which could impact the precision of the estimated variables and subsequently, the interpretations of these estimates. Moreover, the data pertained only to Portuguese soccer players, limiting the generalizability of the findings to players from other nationalities or other sports disciplines.

## Practical Implications

The relationship between the length of the discontinuation stage and the retiring age of soccer players highlights the need for careful career planning throughout a player's active professional career. As players approach the discontinuation stage, proactive steps to prepare for the next phase of their lives may reduce the uncertainty and stress associated with career transitions. Educational programs that equip players with the skills and knowledge to navigate life after soccer could be invaluable in this regard (Fortunato and Marchant, 1999). Furthermore, results may indicate the necessity for mental health support during the discontinuation stage. Sports psychologists could be particularly effective in helping players cope with the psychological challenges of career transition, addressing issues such as identity loss, lack of career direction, and fear of the unknown (Warriner and Lavallee, 2008; Young et al., 2006).

Moreover, the findings underline the role of external influences such as the pressure from teams, fans or personal relationships in extending a player's career beyond what might be personally beneficial or desired. Stakeholders in soccer, including coaches, team managers, and even family members, must be made aware of the potential implications of their influence and should strive to support players in their transition out of the sport (Kerr and Dacyshyn, 2000; Lavallee and Robinson, 2007). To add depth to this interpretation, future research could investigate individual differences in how players experience the discontinuation stage. Factors such as the players' personality, family situation, financial status, and the availability of career transition support could all contribute to the length of the discontinuation stage and the retiring age. Understanding these factors could provide more targeted strategies to support players through their career transitions.

## Conclusions

The findings emphasize the vital role that early career experiences and the management of career discontinuation stages can play in determining when soccer players retire. The significant association between the discontinuation stage length and retiring age underlines the importance of equipping players with the necessary resources to navigate this critical phase in their career trajectory effectively. From a practical standpoint, these findings should prompt the development of comprehensive support programs aimed at facilitating career transitions for soccer players. Such programs should incorporate educational resources, mental health support, and personalized career guidance, enabling players to better manage their transition out of active professional soccer. The results underscore the need for proactive career planning throughout a player's career and the critical role that stakeholders can play in ensuring a smoother transition into retirement.
